# NEUROPSYCHOLOGICAL CONSEQUENCES THREE YEARS POST-COVID-19 ARE PERSISTENT AND HAVE GREAT IMPACT ON EVERYDAY ACTIVITIES

**DOI:** 10.2340/jrm.v58.45473

**Published:** 2026-08-03

**Authors:** Ann BJÖRKDAHL, Jerry LARSSON

**Affiliations:** 1 Sahlgrenska University Hospital, Occupational Therapy and Physiotherapy, Gothenburg; 2 University of Gothenburg, Sahlgrenska Academy, Institute of Neuroscience and Physiology, Rehabilitation Medicine, Gothenburg, Sweden

**Keywords:** post-COVID-19 condition, cognitive dysfunction, fatigue, neuropsychological assessment, long-term outcomes, rehabilitation

## Abstract

**Objective:**

Post-COVID-19 condition (PCC) is associated with persistent cognitive dysfunction and fatigue, but limited evidence is available regarding long-term outcomes beyond one year.

**Design:**

Longitudinal follow-up assessment.

**Patients:**

Patients who had been previously hospitalized or treated in primary care after COVID-19 infection.

**Methods:**

Cognitive performance (WAIS-III subtests, Rey Complex Figure, RAVLT, and D2 Test of Attention) and fatigue (MFI-20 and MFS) were examined in 82 participants at 18 months post-infection; 59 of these participants were reassessed at 36 months. Activity limitations were evaluated using structured interviews.

**Results:**

At 18 months, the scores on most cognitive tests were significantly below expected premorbid levels (mean differences: 7–17 T-points). At 36 months, deficits persisted in most domains, with only WAIS Matrices and D2 KL showing modest improvement (Cohen’s d: ~0.4). Fatigue remained high across all dimensions of MFI-20, and 75–78% of patients exceeding the mental fatigue cut-off of MFS. Self-reported cognitive impact and activity limitations showed minimal change over time.

**Conclusions:**

Post-COVID-19 cognitive dysfunction and fatigue remain prevalent for up to three years post-infection, particularly affecting attention, processing speed, and working memory. These deficits pose substantial barriers to daily functioning and return to work, highlighting the need for targeted rehabilitation strategies.

## LAY ABSTRACT

Many people experience long-lasting problems after COVID-19, even years later. In this study, we followed individuals with prior COVID-19, and evaluated their thinking abilities and fatigue at 18 and 36 months post-infection. We tested memory, attention, and speed of thinking, and asked about tiredness and daily life. Our results showed that most participants still had difficulties with concentration, memory, and mental energy at three years after infection. Fatigue was common, and exhibited little improvement over time. For many participants, these problems made everyday activities and returning to work more difficult. Our findings reveal that COVID-19 can have long-term effects on the brain and energy levels. Additional support and rehabilitation are needed to help people manage these challenges and improve their quality of life.

COVID-19, initially seen as a respiratory illness, is now recognized as a multiorgan disease affecting the central nervous system. Persistent structural and cognitive changes in brain regions linked to olfaction and cognition have been reported (1). A systematic review and meta-analysis describe a pattern of cognitive impairment ranging from 1 to 12 months after COVID-19 illness, across domains, and whether there are predictors of this impairment (2). From 66 studies with a total of 134 different cognitive tests, evidence was found of a global impairment in cognition across the spectrum of COVID-19 disease severity. Both hospitalized and non-hospitalized patients may experience significant residual disabilities for a long period of time (3, 4). The World Health Organization (WHO) has defined a condition with long-lasting problems as “post-COVID-19 condition” (PCC) (5, 6). A Swedish registry study comprising around 40% of the Swedish population (4.1 million individuals) found that 2% of COVID-19 cases were diagnosed as PCC (7). PCC is not restricted to the elderly (8) and is more prevalent in females than males (9). Moreover, there is an overrepresentation among people with higher education (7). Several longitudinal studies show persistent symptoms, especially regarding cognitive dysfunction, in PCC (10–12). Two years after infection, the most common persisting symptoms reported were cognitive dysfunction, sensorimotor difficulties, and fatigue (13). Studies show that cognitive impairment following COVID-19 improves over time, but there is currently insufficient knowledge about whether it fully resolves and, if so, how long that process takes (14). Cognitive impairments appear across cognitive domains without a clear pattern, likely due to heterogeneous studies and methods (2). More research is needed on which cognitive functions are most affected, how fatigue develops over time, its role in cognitive functioning, and the obstacles it poses to everyday activities. This study addresses these gaps.

## AIM

The study’s aim was to investigate the long-term effects of cognitive dysfunction, fatigue, and impact on activity performance following a COVID-19 infection, as well as which cognitive functions are particularly affected.

### Research questions

- What is the status of cognitive function at the 18-month follow-up after contracting COVID-19, relative to the individual’s expected level?
- Which cognitive functions are primarily affected in the study group?
- Do participants experience persistent fatigue at the 18-month follow-up?
- Can improvements in cognitive function and fatigue be observed between 18 and 36 months?
- How do the participants describe the impact on their functional ability at 36 months compared with what was previously described at 12 months?

## METHOD

### Design

The present study is a follow-up study that extends the original study, LECOG-COVID-19, which followed the change in symptoms and activity restrictions in hospitalized and non-hospitalized COVID-19 patients during the first year after onset. The findings from that suggested a need for more extensive neuropsychological testing and extended follow-up time, which is why it was decided to add two follow-ups at 18 and 36 months and a more thorough neuropsychological investigation for the present study.

### Sample

The original study included 122 patients admitted to hospital for a COVID-19 infection (01-07-2020–29-02-2021) and 90 patients admitted to primary care rehabilitation due to rehabilitation needs after a milder acute illness of COVID-19 (01-09-2020–31-08-2021). For the present study patients not of working age or with limitations for participation in neuropsychological tests and interview, such as lack of ability in the Swedish language, were excluded and 104 patients remained and were approached regarding participation in the follow-up. Twenty-two patients declined further participation in the study while 82 individuals consented to participate and were included 18 months after the onset of COVID-19.

## INSTRUMENTS

### Neuropsychological assessments

- *Wechsler Adult Intelligence Scale, WAIS III (15, 16), subtest Information,* measures general knowledge and long-term memory for factual information. It assesses a person’s ability to recall or recognize information concerning common events, objects, places, and people as well as knowledge acquired through education and everyday life experiences.
- *WAIS III, subtest Matrices* was designed to measure non-verbal reasoning abilities. In particular, it assesses: abstract reasoning, problem-solving skills, visual-spatial ability, and cognitive flexibility.
- *WAIS III, Digit span subtest* measures short-term auditory memory and working memory including attention, concentration, and cognitive flexibility. The test involves two parts: Digit span forward, and Digit span backward.
- *WAIS III, Symbol coding subtest* measures processing speed and involves visual-motor coordination, attention, concentration, and short-term visual memory. The test assesses cognitive efficiency.
- *WAIS III, Arithmetic subtest* measures quantitative reasoning and working memory. Specifically, it evaluates: numerical reasoning, working memory, attention, concentration, problem-solving skills, and cognitive flexibility. The participant is presented with a series of mathematical problems that must be solved mentally within a time limit.
- *REY-Osterreith Complex figure test* (ROCF) is a test used to evaluate several cognitive functions (17). The primary areas measured are: visual-spatial construction ability, visual memory, planning and organizational skills, attention and concentration, and executive function.
  *ROCF A* – copying the figure, *ROCF B* – reproduction of the figure from memory (immediate recall), *ROCF C* – drawing again from memory after 20–30 min (delayed recall).
- *D2 Test of Attention* is an assessment of selective attention and concentration (18, 19). It evaluates how well a person can focus on relevant stimuli while ignoring irrelevant ones. The test is used to assess attention span, processing speed, and the ability to maintain focus over time.
  Key components of the test are:
  - *F%* (error percentage), reflecting accuracy.
  - *BZO* (error-corrected target rate) measuring the speed of correct responses calculating the number of correctly identified targets minus the number of errors. It indicates the person’s ability to correctly process information while maintaining concentration.
  - *KL* (Concentration performance) produce a concentration score representing the number of correct target selections minus the commission errors (incorrect selections). It gives an indication of how well the individual can focus on relevant stimuli while avoiding distractions.
- *The Rey Auditory Verbal Learning Test (RAVLT)* evaluates verbal memory and learning abilities and measures Verbal learning, Short-term memory, Long-term memory, Susceptibility to interference, and Recognition memory (20). The test involves presenting a list of 15 unrelated words over multiple trials. After each trial the participant is asked to recall as many words as possible. The test includes several phases:

Questionnaires:

- *Multidimensional Fatigue Inventory 20, MFI-20*, is a 20-item questionnaire evaluating 5 dimensions of fatigue: general fatigue, physical fatigue, reduced motivation, reduced activity, and mental fatigue. Responses are rated on a 5-point scale (1 = “yes, that is true” to 5 = “no, that is not true”) indicating how aptly certain statements regarding fatigue represent their experiences. Total scores range from 20–100 and the dimension total score is from 4–20. Higher total scores correspond with more fatigue (21, 22).
- *Mental Fatigue Scale, MFS* is a 15-item questionnaire on mental fatigue. Each question is followed by 4 statements that describe: No (0), Slight (1), Fairly serious (2), and Serious (3) problems. There are also figures to indicate an answer between 2 statements. If the sum score exceeds 10.5 points this indicates problems with brain fatigue (23).
- *Cognitive Failure Questionnaire, CFQ*, is a self-report measure to assess the frequency of lapses of attention, memory, and cognition in everyday life consisting of 25 questions concerning “minor mistakes that may occur”. CFQ is rated from 0 (never) to 4 (very often), and items are summed to a total score (0–100) and can be divided into the categories: forgetfulness, distractibility, and false triggering (24).

### Implementation and data collection

18 months after the onset of COVID-19 the participants were invited for neuropsychological testing and a short interview by a psychologist specialized in neuropsychology. The testing took between 1 and 1.5 h including a short interview on education, employment, and kind of work they performed. Questionnaires were sent by link on email. If necessary, reminders were sent.

At the 36-month follow-up, the same procedure with testing and interview as at 18 months was carried out. However, at this time the interview was extended and conducted by an occupational therapist in the same way as was the case for the original study at 12 months to enable exploration of activity limitations that are likely to be closely related to the existing impairments in cognition and fatigue. The interview at 36 months included a reflection on the same areas as at the 12 months interview: cognitive dysfunction, personal care, household, leisure and work, and eventual changes since the 12-month interview in the original study. All the interviews were conducted by the same occupational therapist using a guide with the interview areas to discuss and the responses given at the 12-month interview to be able to reflect on changes between the 2 time points. The occupational therapist read aloud what the participant had said during the previous session and asked them to describe what remained the same or had changed.

On both occasions (12 and 36 months), the occupational therapist made an estimate from the answers in the interview of the degree of problems in the following way: not at all (0), some (1), and major (2) problems. Comparison was made between the two occasions to explore the change over time. At 36 months a general question regarding their recovery, until 36 months, was posed with the following response alternatives: recovered (3), improved (2), some improvement (1), and no improvement (0).

The tests were corrected, and raw scores and *t*-values were entered into SPSS version 28.0.1.1 (IBM Corp, Armonk, NY, USA). Responses from forms in Esmaker were imported into the same file.

### Data analysis

Descriptions of the sample were made regarding age, gender, education, hospitalization or not, and pandemic wave at onset. Descriptive of all tests and questionnaires, at 18 and 36 months, were given in tables including mean (standard deviation, SD), and median (min–max). Nominal data were presented as percentages. The extent of remaining problems in activity performance was described as proportions of the estimations of no, some, and major problems in each activity area. Presence or not of cognitive deficits was described as percentage yes or no. The general question on recovery was described as proportions in each category (recovered, improved, some improvement, and no improvement). Comparisons between 12 and 36 months regarding the levels of problems in the different activity areas were performed using a χ^2^ test.

To produce values of premorbid level the “hold” method was used, which makes use of performances on tests that are resistant to neurological damage. The difference between “hold value” and the actual results of the various tests provided a reasonable estimate of deterioration compared with expected performance before contracting COVID (25). This approach is well supported psychometrically (26) and was chosen as the sample consisted of highly educated individuals with an expected premorbid level above the norm. The results from WAIS III Information were used for estimation of the premorbid level. The test was chosen as WAIS information reflects crystallized knowledge, which tends to be stable over time and is not as sensitive for ageing or neurological deficits as fluid intelligence and therefore may predict the premorbid level (27–29). The neuropsychological test battery used was selected to cover a range of areas that could be expected to be affected by the disease. *T*-values were used for the results of the neuropsychological tests, which were calculated from the raw scores of the various tests; these scores are adjusted for age and gender. The analyses made were paired *t*-tests between expected value (WAIS III information) and each of the tests to explore whether the actual results from 18 and 36 months differ significantly from expected. Mean differences of the tests have been counted and are given in Table III (see Results). The mean of the normal population is T50 (SD 10) and a typical variation for an individual on different subtests is around ± 5–7 T-values (½ to ¾ SD), differences of 10 between two subtests occur, but are not the majority, and differences of 15–20 T-values (1.5–2 SD) are uncommon (30). However, such variability is typically unsystematic. Therefore, consistent domain-specific differences observed at the group level are unlikely to reflect normal variation alone and may indicate selective cognitive inefficiency.

Analyses of change over time from 18 to 36 months were made by *t*-test for paired samples including both neuropsychological tests and total scores from questionnaires. Effect size was given as Cohen’s *d* as follows: *d* = 0.2 small effect, *d* = 0.5 moderate effect, and *d* = 0.8 large effect.

Significance was set to *p* < 0.05. The aspect of mass significance when performing many tests was considered. Due to the clear significance (< 0.001) on the comparisons of actual and expected values on test no adjustment was made. Regarding changes over time that were not so clear, corrections with Bonferroni–Holm were performed.

### Data from earlier analysed qualitative data

To support the discussion of the significance of results with reduced ability in neuropsychological tests and questionnaires on fatigue, the results from a qualitative analysis of interviews at 12 months in a previous article describing obstacles to work was reused (31). The sample used was consistent with that in the current study.

The previous qualitative analysis from 12 months resulted in 6 categories; two of them related to cognitive impact (“Reduced cognitive ability” and “Generally reduced ability to carry out work tasks”), and two categories reflected fatigue (“lack of energy” and “decreased mental stamina”) (31). The subcategories of the category “Reduced cognitive ability” were: “Decreased ability to concentrate”, “Difficulty in multitasking”, “Risk of forgetting information and agreements”, “Difficulty to cope with distractions” and “Difficulty to solve problems and draw conclusions”. The categories regarding fatigue included aspects of reduced tempo, lower quality in activity performance, lack of initiative, difficulties with stimuli-rich environment and risk for overload, and that the brain “shut down”. The category “Generally reduced ability to carry out work tasks” consisted of the subcategories “Need of support from others”, “Limited ability to perform certain tasks such as leadership”, “Difficulties to manage meetings with several people involved”, “Difficulty to fully hold in work that involves customer contact”, “Difficulty to take notes at meetings”(31).

The two additional categories comprising the qualitative result (“Decreased physical capability to work” and “Lack of understanding and support”) were not found to be relevant for the discussion regarding the results of the present study, which has a focus on cognition and fatigue (31).

## RESULTS

The sample consisted of 82 individuals at 18 months willing to participate again after the 12-month follow-up (*n* = 91). At the 36-month follow-up 59 remained. The dropout to 36 months was due to 12 individuals not wanting to participate, 3 had moved, 5 were unreachable, and 3 had other illnesses that prevented participation. The sample consisted of equal proportions of men and women. Most of the participants had fallen ill during the first and second pandemic wave and 63% had not been hospitalized. Most of the sample had higher education. At 36-month follow-up 43% had returned to work full time and 19% had not been able to return at all (Table I).

**Table I:** Description of the sample

Variable	Unit	Result
**Age** (*n* = 82)	Mean (SD)	49.52 (11.64)
**Gender** (*n* = 82)	Men/Women	47.6%/52.4%
**Education** (*n* = 82)	Primary and lower secondary Upper secondary /high school Higher education	*n* = 4 (4.9%) *n* = 21 (25.6%) *n* = 57 (69.5%)
**Severity at onset** (*n* = 82)	Hospitalized Non-hospitalized	*n* = 30 (36.5%) *n* = 52 (63.5%)
**Pandemic wave**	I Mar 2020–Sept 2020 II Oct 2020–Jan 2021 III Feb 2021–Jun 2021	*n* = 36 (44%) *n* = 27 (33%) *n* = 19 (23%)
**Employment status** at 36 months (*n* = 53)	0% 25% 50% 75–85% 100%	*n* = 10 (18.9%) *n* = 5 (9.5%) *n* = 9 (17.0%) *n* = 6 (10.3%) *n* = 23 (43.4%)
**Hospital days (total days)** Included from hospital (*n* = 20) Including primary care + earlier hospital (*n* = 10)	Mean/median/SD/min–max Mean/median/SD/min–max	33.8/15.5/39.4/6–175 18.4/18/11.8/3–40
**Hospital Intensive care (days)** Included from hospital (*n* = 20) Including primary care + earlier hospital (*n* = 10)	Mean/median/SD/min–max Mean/median/SD/min–max	24.4/18/27.6/4–103 15.5/12.5/9/6–29

### Neuropsychological tests at 18 months

**Fig. 1:**
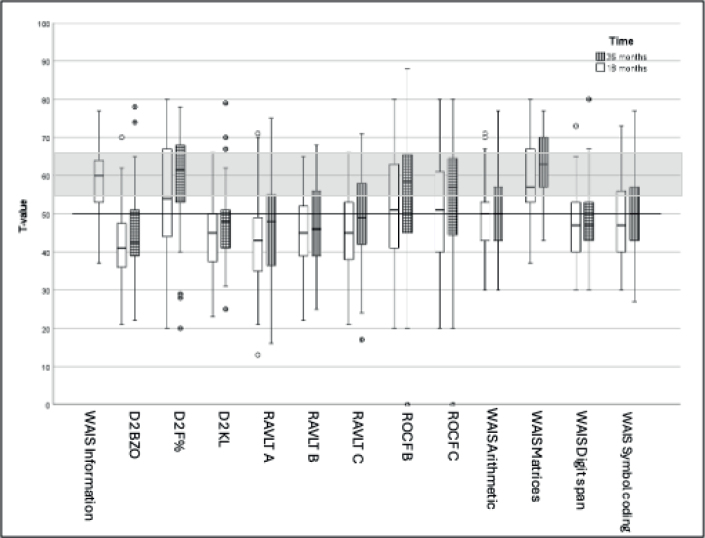
Boxplots of the results of the different neuropsychological tests included in the study at 18- and 36-month follow-up. The boxes represent 50% of the sample. The whiskers extend to the smallest and largest value except for outliers that are marked. The bold line in the middle of the figure indicates the value T50 which is the normative population mean. The grey area indicates expected mean±1 SD for the study sample.

The results of all the neuropsychological tests at 18 and 36 months are presented as box plots in Fig. 1. The figure also shows the area in grey where most of the results were expected to be in this sample (sample mean of hold value ± 1 SD), indicating that the sample had an expected premorbid level of T59, almost 1 SD above average in the population (norm mean T50 ± 1 SD).

At 18 months, 10 of the 12 included neuropsychological tests showed significantly lower results (*p* < 0.001) than expected (Table II). The mean difference from expected ranged from 7–17 T-values. (“A typical variation for an individual on different subtests is around ± 5–7 T-values [½ to ¾ SD], differences of 10 between two subtests occur, but are not the majority, and differences of 15–20 T-values [1.5–2 SD] are uncommon” [30].) The non-significant tests at 18 months were matrices that were even somewhat better than expected and D2 F% also gave a result close to the expected.

**Table II:** Neuropsychological test performance reported as *t*-values, deviations from expected performance, and between-time-point comparisons with *p*-values and effect sizes

Instrument	*t*-values	18 months *n* = 82	Difference from expected value 18 months	*p*-value of difference from the expected value at 18 months	36 months *n* = 59	Difference from expected value 36 months	p-value of difference from the expected value at 36 months	Change between 18 and 36 months		Effect size temporal change
Predicted premorbid level WAIS Information	Mean (SD) Median (min–max)	58.31 (8.48) 60.00 (37–77)						Nominal *p*-value	Adjusted *p*-value	Cohen’s *d* 95% CI
REY complex figure B	Mean (SD) Median (min–max) % below 1 SD of exp	51.21 (15.86) 51 (20–80)	7.01 (15.75) 7 (–42 – 28) 40%	*p* < 0.001 * 95% CI 3.48;10.54	55.12 (16.63) 58.5 (0–88)	2.86 (15.12) 0 (–64 – 28) 31%	*p* = 0.155 95% CI –1.11;6.83	*p* = 0.295 95% CI (–5.35; 1.65)	*p* = 591	d = –0.137 95% CI (–0.39; 0.12)
REY complex figure C	Mean (SD) Median (min–max) % below 1 SD of exp	49.56 (15.70) 51 (20 – 80)	8.78 (15.56) 9 (–44 – 28) 46%	*p* < 0.001* 95% CI 5.29;12.27	53.88 (16.66) 57 (0 – 80)	4.35 (15.08) 2 (–64 – 28) 30%	*p* < 0.034* 95% CI 0.348;8.36	*p* = 0.102 95% CI (–6.56; 0.61)	*p* = 308	*d* = –0.218 95% CI (–0.477; 0.04)
RAVLT A	Mean (SD) Median (min–max) % below 1 SD of exp	43.12 (11.92) 43 (13 – 71)	15.18 (10.78) 15 (–36 – 6) 64%	*p* < 0.001* 95% CI 12.77;17.57	46.31 (13.20) 48 (16 – 75)	12.28 (11.53) 11 (–37 – 12) 51%	*p* < 0.001 * 95% CI 9.24;15.31	*p* = 0.047 95% CI (–5.12; –0.29)	*p* = 0.285	*d* = –0.264 95% CI (–0.52; –0.00)
RAVLT B	Mean (SD) Median (min–max) % below 1 SD of exp	44.99 (10.55) 45 (22 – 65)	–13.33 (10.93) 12.5 (–41 – 8) 54%	*p* < 0.001* 95% CI 10.89;15.76	47.68 (10.34) 46 (25 – 68)	10.98 (10.54) 11 (–39 – 11) 51%	*p* < 0.001 * 95% CI 8.20;13.75	*p* = 0.016 95% CI (–4.83; –0.52)	*p* = 0.127	*d* = –0.323 95% CI (–0.58; –0.06)
RAVLT C	Mean (SD) Median (min–max) % below 1 SD of exp	45.63 (10.34) 45 (21 – 66)	12.65 (9.75) 11 (–36 – 11) 56%	*p* < 0.001* 95% CI 10.47;14.82	48.63 (11.30) 49 (17 – 71)	10.19 (10.46) 9 (–36 – 14) 43%	*p* < 0.001 * 95% CI 7.43;12.94	*p* = 0.009 95% CI (–5.31; –0.784)	*p* = 0.092	*d* = –0.351 95% CI (–0.61; –0.085)
WAIS Matrices	Mean (SD) Median (min–max) % below 1 SD of exp	58.82 (9.86) 57 (37–80)	–0.91 (9.79) 0 (–20 – 24) 11%	*p* < 0.407 95% CI –3.09;1.26	62.40 (7.65) 63 (43 – 77)	–3.72 (9.52) –3 (16 – 26) 5%	*p* < 0.005 * 95% CI –6.24;–1.19	*p* = 0.002 95% CI (–4.98; –1.12)	*p* = 0.029*	*d* = –0.415 95% CI (–0.68; –0.14)
WAIS Arithmetic	Mean (SD) Median (min–max) % below 1 SD of exp	48.69 (9.10) 50 (30 – 71)	9.51 (8.95) 8.5 (–30 – 10) 44%	*p* < 0.001* 95% CI 7.52;11.50	51.38 (10.58) 50 (30 – 77)	7.07 (9.47) 7 (–31 – 10) 36%	*p* < 0.001 * 95% CI 4.55;9.58	*p* = 0.071 95% CI (–3.27; 0.14)	*p* = 0.356	*d* = 0.090 95% CI (–0.34; 0.18)
WAIS Symbol Coding	Mean (SD) Median (min–max) % below 1 SD of exp	48.28 (10.11) 47 (30 – 73)	9.65 (11.79) 10. (–34 – 20) 50%	*p* < 0.001* 95% CI 7.02;12.27	49.52 (9.85) 50 (27 – 77)	8.71 (11.90) 10 (–37 – 17) 41%	*p* < 0.001 * 95% CI 5.57;11.83	*p* = 0.357 95% CI (–3.26; 1.19)	*p* = 0.357	*d* = –0.120 95% CI (–0.37; 0.13)
WAIS Digit Span	Mean (SD) Median (min–max) % below 1 SD of exp	46.91 (8.22) 47 (30–73)	11.09 (9.67) 10.5 (–37 – 13) 50%	*p* < 0.001* 95% CI 8.93;13.24	49.17 (9.03) 47 (30 – 80)	9.31 (9.71) 10 (–30 – 14) 43%	*p* < 0.001 * 95% CI 6.75;11.86	*p* = 0.024 95% CI (–2.92; –0.21)	*p* = 0.172	*d* = –0.298 95% CI (–0.55; –0.03)
D2 Test of attention, F%	Mean (SD) Median (min–max) % below 1 SD of exp	55.13 (14.36) 54 (20 – 80)	2.83 (14.31) 3 (–40 – 40) 19%	*p* < 0.084 95% CI –0.39;6.06	59.02 (12.18) 61.5 (20 – 78)	–0.21 (15.47) –3 (–44 – 30) 19%	*p* < 0.898 95% CI –4.37;3.84	*p* = 0.078 95% CI (–6.78; 0.36)	*p* = 0.310	*d* = –0.238 95% CI (–0.50; 0.026)
D2 BZO	Mean (SD) Median (min–max) % below 1 SD of exp	41.34 (9.81) 41 (21 – 70)	13.96 (10.20) 13 (–40 – 21) 65%	*p* < 0.001* 95% CI 14.36;19.48	44.86 (11.16) 42.5 (22 – 78)	13.81 (12.73) 15 (–40 – 25) 65%	*p* < 0.001 * 95% CI 10.42;17.18	*p* = 0.012 95% CI (–6.12; –0.79)	*p* = 0.108	*d* = –0.344 95% CI (–0.61; –0.075)
D2 KL	Mean (SD) Median (min–max) % below 1 SD of exp	44.24 (8.61) 45 (23 – 66)	16.92 (11.36) 17.5 (–41 – 15) 58%	*p* < 0.001* 95% CI 11.66;16.26	47.69 (9.70) 48 (25 – 79)	11.12 (11.77) 12 (–38 – 17) 58%	*p* < 0.001 * 95% CI 7.99;17.85	*p* = 0.004 95% CI (–5.79; –1.15)	*p* = 045*	*d* = –0.397 95% CI (–0.66; –0.12)

* Indicates significance < 0.05.

At 18 months, RAVLT A (64%), D2 BZO (65%), and D2 KL (58%) were the tests in which most participants had a score more than 1 SD below the expected (Table III).

**Table III:** Mean deviation from expected values at 18-month follow-up in each of the included subtests and variation in the material

Subtest	Mean difference from expected	Min	Max	SD
D2 BZO	–16.92	–41	15	11.36
RAVLT A	–15.17	–36	0	10.78
D2 KL	–13.96	–40	21	10.2
RAVLT B	–13.32	–41	8	10.93
RAVLT C	–12.65	–36	11	9.75
Digit span	–11.08	–37	13	9.67
Symb/code	–9.65	–34	20	11.79
Arithmetic	–9.51	–30	10	8.95
ROCF C	–8.78	–44	28	15.56
ROCF B	–7.02	–28	42	15.75
D2 F%	–2.8	–40	40	14.31
Matrices	0.91	–20	24	9.79

### Neuropsychological tests at 36 months

The differences between actual results and the expected were still significant in 10 of 12 tests at 36 months. At 36 months the two tests with non-significant results were ROCF B and D2F%. The result of Matrices was at 36 months significantly higher than expected (*p* = 0.005). Due to the clear significance of these results, they were not adjusted for mass significance.

At 36 months, RAVLT A (51%), D2 BZO (65%), and D2 KL (58%) were the tests in which most participants had a score more than 1 SD below the expected (see Table III). Among the participants, the number of tests on which they had unexpectedly low results varied (0–11 tests). Difficulties with 7–8 tests were common but so were expected results on all but 3 tests (Table IV).

**Table IV:** Number of participants by count of neuropsychological tests with performance ≥ 1 SD below expected value (0–12 tests)

Number of tests with results ≥ 1 SD below the expected	Number of participants
0	3
1	5
2	5
3	11
4	6
5	6
6	5
7	10
8	13
9	6
10	8
11	2
12	0

### Change between 18 and 36 months

Significant change over time from 18 to 36 months was found in 7 of the tests and not as clear significance as the analyses between expected and actual results on tests, which is why a Bonferroni–Holm adjustment was undertaken. After that only two tests still showed a significant difference to the better between the time points, the WAIS Matrices (*p* = 0.029) and D2 KL (*p* = 0.045). The effect sizes on the tests were small according to Cohen’s *d* (0.20–0.49) (see Table II), with the effect size of *d* = 0.41 for WAIS Matrices and *d* = 0.39 for D2 KL.

### Power of the study

The study included 82 participants. The sample size was determined by the number of individuals available from the original study, as this investigation represents a follow-up of a predefined subgroup and the sample size could therefore not be influenced by the present study. Based on standardized T-values (SD 10) and a two-sided significance level of 0.05, this sample size is generally sufficient to detect moderate-to-large effects with adequate statistical power. The observed group differences of 7–16 T-values correspond to large effect sizes (approximately Cohen’s *d* = 0.7–1.7), suggesting that the study had adequate power to detect the observed effects.

### Cognitive Failure Questionnaire

The results from the questionnaire CFQ regarding the self-reported frequency of lapses of attention, memory, and cognition in everyday life corresponded well to the objective neuropsychological tests with an affected performance for most participants without positive change between 18 and 36 months (Table V). The type of lapses reported were equally distributed between the three different categories: forgetfulness, distractibility, and false triggering. The perceived cognitive impact expressed in the interviews at 36 months was great, with 77% of the sample experiencing some or great impact and the percentage was almost identical to their experience at 12 months (32).

**Table V:** Outcome from questionnaires at 18 and 36 months and interviewed-based ratings of persistent cognitive difficulties and perceived change between 12 and 36 months

Instrument		18 months *n* = 62	36 months *n* = 58	Change over time
MFI-20 total score	Mean (SD) Median (min–max)	65.36 (19.32) 66.5 (24–98)	66.05 (17.36) 65 (29–96)	*p* = 0.224 95% CI –1.91–7.95
MFI-20 General fatigue	Mean (SD) Median (min–max)	14.28 (2.53) 14.5 (8–20)	14.54 (2.67) 15 (9–19)	*p* = 0.836 95% CI –0.85–0.69
MFI-20 Physical fatigue	Mean (SD) Median (min–max)	13.62 (4.82) 14 (5–20)	13.47 (4.37) 14 (5–20)	*p* = 0.327 95% CI –0.59–1.74
MFI-20 Reduced activity	Mean (SD) Median (min–max)	13.13 (4.85) 12 (5–20)	13.37 (4.46) 14 (5–20)	*p* = 0.318 95% CI –0.68–2.05
MFI-20 Reduced motivation	Mean (SD) Median (min–max)	10.35 (4.04) 10 (4–20)	10.54 (3.82) 9 (4–20)	*p* = 0.684 95% CI –0.97–1.47
MFI-20 Mental fatigue	Mean (SD) Median (min–max)	13.06 (4.17) 14 (4–20)	12.96 (3.96) 13 (8–20)	*p* = 0.066 95% CI –0.05–1.74
MFS total score	Mean (SD) Median (min–max)	16.32 (7.89) 15.75 (0–33)	16.47 (7.69) 16.5 (0.5 – 36)	*p* = 0.337 95% CI –0.79–2.28
MFS cut-off 10.5	% above cut-off	75	78	
CFQ total score	Mean (SD) Median (min–max)	67.23 (17.48) 66 (33 –115)	70.46 (15.23) 70 (44 – 105)	*p* = 0.229 95% CI –6.05–1.48
CFQ Forgetfulness	Mean (SD) Median (min–max)	24.86 (6.54) 25 (12 – 39)	25.88 (5.44) 25 (17 – 39)	*p* = 0.201 95% CI –1.93–0.41
CFQ Distractibility	Mean (SD) Median (min–max)	21.86 (5.61) 21 (11 – 35)	22.67 (5.17) 22 (12 – 39)	*p* = 0.470 95% CI –1.91–0.89
CFQ False triggering	Mean (SD) Median (min–max)	18.65 (6.04) 18.5 (9 – 35)	19.67 (5.48) 19.5 (11 – 34)	*p* = 0.407 95% CI –1.75–0.71
Perceived cognitive impact	% None Some Great	12 months 23.8 49.2 27.0	36 months 22.4 51.7 25.9	
Perceived change in impact of PCC from 12 to 36 months	% no improvement % slightly improved % improved % recovered		28.1 40.4 21.1 10.5	

^a^ Group means of sum scores are reported for the questionnaires and between-time-point comparisons at 18 and 36 months with *p*-values and confidence intervals. ^b^ Interview data are expressed as percentages within response categories.

### Perceived dysfunction reported in interviews

The experience of dysfunction reported in interviews at 12 months remained largely unchanged in terms of both symptoms and activity capacity (Table V and VI). Based on this, the qualitative analysis of interviews and the categories that emerged at 12 months are considered relevant as a basis for discussion of the various neuropsychological test results and their relationship to limitations in activity capacity at 36 months (31).

**Table VI:** Occupational therapist’s ratings of problems across activity domains at 12 and 36 months

Activity areas	Extent of problems	12 months		36 months	
		*n*	%	*n*	%
Personal care	No problems	35	58.7	39	68.4
	Some problems	17	28.6	16	28.1
	Great problems	6	12.7	2	3.5
Household	No problems	16	27.0	20	35.1
	Some problems	21	36.5	24	42.1
	Great problems	21	36.5	13	22.8
Leisure	No problems	8	15.6	16	28.1
	Some problems	23	37.5	20	35.1
	Great problems	27	46.9	21	36.8
Work	No problems	17	29.8	15	26.8
	Some problems	12	21.1	18	32.1
	Great problems	28	49.1	23	41.1

^a^Problem severity was rated by occupational therapist as none, some, or great for the domains personal care, household, leisure, and work. ^b^Data are presented as number of participants (*n*) and percentage within each severity category.

### Fatigue

The analyses of the questionnaires on fatigue showed a high prevalence of fatigue (MFI-20, MFS) at 18 months that was still present at 36 months, and no significant change was detected (see Table V). For the domains of MFI-20, they all had similarly high scores of fatigue in different domains on both occasions, except for the domain “reduced motivation”, which was somewhat lower than the other domains. On the MFS, 75% and 78% of the sample at 18 and 36 months respectively had a score above the cut-off of 10.5 points, indicating problems with brain fatigue affecting the participants’ life. No improvement was seen between 18 and 36 months (see Table V).

### Activity performance

Table VI indicates the occupational therapist`s ratings of problems in the different activity areas at 12 and 36 months. From the table it can be seen that in all areas except for personal care there were still many participants experiencing problems after 36 months especially regarding work and leisure. However, the comparisons between the two occasions showed significant change in all four areas (personal care *p* = < 0.001, household p = < 0.001, leisure *p* = < 0.001, work *p* = 0.003) towards fewer problems at 36 months.

The questions at 36 months regarding improvements in activity performance since the 12-month interview confirmed the results from the tests and questionnaires that deficits persisted, and the improvements were scarce (see Table V). Only 10.5% felt they had recovered and as many as 28% perceived that they were not improved at all.

## DISCUSSION

The results showed high levels of fatigue at both follow-ups (18 and 36 months) without any significant change between timepoints. The sample was well-educated and had an expected level on the neuropsychological tests almost 1 SD above the normal population. Around half of the sample performed more than 1 SD below the expected level at 18 months. By 36 months, performance had improved slightly but remained below the expected level with around 77% of the sample still experiencing some or great cognitive impact. The results are consistent with other research showing a significant cognitive impact in PCC and as much as an 18-fold greater risk of cognitive impact than in individuals who have not been infected (33). Research findings also indicate that the symptoms of cognitive dysfunction are those most persistent over time (4, 34, 35) and that also after 2 years the performance on standardized neuropsychological testing was not within normative parameters (36). Identified neuropsychological manifestations of COVID-19 can significantly impede functioning and may decrease productivity and quality of life (33).

There are many studies reporting cognitive deficits after COVID-19 infection and commonly cognitive screening measures such as the Montreal Cognitive Assessment (MoCA) were employed. Cognitive screening instruments give a rough measure of global cognition without taking age and education into account to any great extent. The use of screening instruments with cut-off scores can therefore give a picture that does not correspond to the individual’s experience of obstacles to performing the various everyday activities they usually do, as the results are above the cut-off (32).

The present study therefore aimed at giving a more detailed picture of the extent of impact and in which areas cognitive dysfunction appears. A test battery with a total of 13 well-validated neuropsychological subtests, including WAIS III information, was used. The choice of tests was based on earlier research and the type of obstacles that were described in the interviews at the 12-month follow-up. From the literature we found that the WAIS III information subtest could be suitable to predict premorbid level of cognitive function as being a test of crystalized intelligence (28, 37, 38). The “hold” method was used, which makes use of performances on tests that are resistant to neurological damage. The difference between “hold value” and the actual results of the various tests provided a reasonable estimate of deterioration compared with expected performance before contracting COVID-19 (25). This approach is well supported psychometrically (26) and was chosen because the sample consisted of highly educated individuals with an expected premorbid level above the norm.

It can be challenging to identify a cognitive deficit in high-functioning individuals because they are much less likely to obtain low test scores, as a much greater change in functioning needs to occur before they perform one or more SDs below the normative mean (39). Brooks et al. (40) demonstrated that isolated low scores, or even a small number of low scores, are common in healthy individuals when multiple neuropsychological tests are administered. However, the presence of low performance across most test measures is uncommon in healthy populations and suggests a pattern of cognitive dysfunction rather than normal variation in test performance (40). As the results of the current study showed that participants in many cases (48%) had unexpectedly low scores (>1 SD below expected) in as many as 7–11 tests, it can be said with relative certainty that there was a clear decline in cognitive function after contracting COVID-19. This was also in line with the participants’ perceptions.

Included tests can be categorized according to the main abilities tested in each subtest. The categories used were verbal comprehension (WAIS III info), working memory (WAIS III Arithmetic and Digit span), learning and recall (RAVLT, ROCF B+C), perceptual organization (WAIS III Matrices), structural planning (ROCF A+B+C), and attention and speed of processing (D2 BZO and KL, WAIS III symbol/coding). Only two of the tests had results within the expected range at 18 months and one of them was D2 F% that measures accuracy, which could be attributed to personal characteristics, perhaps reflecting the current sample of high-functioning individuals. Expected results were also found in WAIS III Matrices at 18 months and ROCF B at 36 months, where ample time was given, and different response options are visible throughout the task. One of the consequences after COVID-19 infection shown in the literature is a reduction in processing speed (41, 42). Matrices mainly require logical ability and perceptual organization. The result on Matrices was almost exactly the expected result at 18 months and at 36 months the positive significant difference was probably due to more alertness and energy to concentrate on the task. To do well on the ROCF test, you need to be able to see the whole picture and plan how to draw it. If this is well done at ROCF A copying, it facilitates the reproduction at B and C. Common for matrices and ROCF are fewer demands on speed and more emphasis on crystallized intelligence and earlier experiences. An explanation for a lower result on these two tests at 18 months could be fatigue, with not enough energy to make the planning (ROCF) and process the information properly (43). RAVLT A, D2 BZO, and D2 KL were the tests with the highest underperformance. All three tests require attention, working memory, concentration, and processing speed. In addition to this, RAVLT A sets requirements for learning ability.

These mainly affected tests can be seen as the control functions that are part of executive function and needed for fluid intelligence. The control functions involve the ability to focus on relevant information and ignore distractions, to hold and manipulate information for short periods of time, and to maintain concentration over time despite distraction or fatigue. In the study interviews, our participants expressed that they had decreased ability to concentrate, to cope with distractions, solve problems, and draw conclusions (31), which is completely in line with the test findings. The participants described how this cognitive dysfunction caused difficulties at work, such as taking notes at meetings, carrying out tasks involving customer contact, and continuing in a supervisory or managerial role (31). The present study comes to a similar conclusion to other research, i.e., that the most prominent post-COVID-19 neuropsychological deficits are difficulties in memory and learning, attentional processes, executive functions, and processing speed (33).

The questionnaires on fatigue in the present study showed a high prevalence of fatigue (MFI-20, MFS) at 18 months, which was still present at 36 months without significant change over time. Fatigue is one of the most persistent symptoms of PCC, which may be present in up to 80% of patients (13). Post-COVID-19 fatigue syndrome associated with subjective cognitive impairment has shown changes in brain functional activity in the areas connected with information processing speed and quality (44) in line with our findings on neuropsychological tests and the reporting of fatigue. From the qualitative analyses of interviews of the same sample at 12 months, the categories regarding fatigue included aspects of reduced tempo, lower quality in activity performance, lack of initiative, difficulties with stimuli-rich environment, and risk of overload and that the brain “shut down” (31).

An earlier article from our group on return to work with the present sample described that, at 12 months, 70% of the hospital care group were back at full-time work while only 50% in the primary care group with milder onset were working full time. In both groups 20% had still not returned to work at all at 12 months (31). An important obstacle for returning to work was the experience of reduced cognitive ability causing problems with concentration, multitasking, remembering, coping with distractions, solving problems, and making conclusions. This meant a need for support from others in the workplace, difficulty in coping with leadership roles, handling meetings with several people, taking notes, and maintaining customer contact (31). The pattern of cognitive dysfunction observed in the present study provides a plausible explanation for the work-related activity limitations previously reported in this cohort, as deficits in attention, working memory, and processing speed are essential for managing complex cognitive demands at work.

The study has limitations but also strengths. A major limitation of the study is that we do not have premorbid data from neuropsychological tests. To try to compensate for this and estimate premorbid levels, the study used the Hold method. This is a well-established method that hopefully provided relevant information and thus still allowed us to demonstrate changes due to the illness that would otherwise not have been visible if we had used only normal values for this highly educated group. This could therefore be seen as a strength of the study as we demonstrate that the perceived decline in cognitive ability is also objectively reflected in test results. Another factor that may have played an limitating role is that some of the subtests used are not the latest version, as we used the WAIS III. Because we have only conducted analyses in which the participants served as their own controls, we can conclude that this should not have a significant impact. Another strength of the study is the connection that can be drawn within the group between quantitative and qualitative results, which provide insight into how cognitive impairments and fatigue affect daily life and work.

In summary, the study shows that after as long as 3 years, there are still significant limitations in terms of cognitive ability and fatigue, which greatly restrict participants in their everyday and working lives. The study also provides an understanding of which cognitive aspects are limited and how these are perceived as obstacles in the work situation. Based on these results, there is a far-reaching remaining need for rehabilitation. It is also important that authorities understand these limitations to handle them fairly, and that employers gain knowledge of the problem in order to be able to make appropriate adjustments.
